# Developing glycoproteomics reveals the role of posttranslational glycosylation in the physiological and pathological processes of male reproduction

**DOI:** 10.1002/imo2.10

**Published:** 2024-07-01

**Authors:** Qingyuan Cheng, Mengqi Luo, Zihe Xu, Fuping Li, Yong Zhang

**Affiliations:** ^1^ Department of Nephrology, Frontiers Science Center for Disease‐Related Molecular Network, West China Hospital, Institutes for Systems Genetics Sichuan University Chengdu China; ^2^ Department of Andrology/Sichuan Human Sperm Bank West China Second University Hospital, Sichuan University Chengdu China; ^3^ Key Laboratory of Birth Defects and Related Diseases of Women and Children, Ministry of Education Sichuan University Chengdu China

**Keywords:** glycosylation, male infertility, male reproduction, semen glycoproteomics

## Abstract

Glycosylation plays a pivotal role in the physiological and pathological processes of male reproduction. It impacts thousands of proteins and is actively ongoing through all stages of reproduction, including spermatogenesis, maturation, capacitation, and fertilization. However, our grasp on glycosylation within male reproductive processes remains limited, largely due to the technical hurdles. Recent advancements have seen the mapping of the glycoproteome of human semen, utilizing cutting‐edge glycoproteomic technologies. This breakthrough lays the groundwork for in‐depth research into the influence of glycosylation on male reproductive system and related disorders. Nevertheless, the field faces numerous challenges that necessitate further advancements in glycoproteomic methodologies. In this analysis, we evaluated the potential applications of advanced glycoproteomic techniques in the study of male reproduction and summarized the detailed profiling of the human semen glycome and glycoproteome. Our current understanding of glycosylation's role within the male reproductive system alongside recent progress in glycoproteomics may equip biologists with a comprehensive insight. Furthermore, this analysis brought together findings on abnormal glycosylation and its link to male reproductive disorders in the view of glycomics and glycoproteomics. It can facilitate the clinical application of glyco‐related biomarkers and targets in the treatment of infertility.

## Highlights

- The understanding of glycosylation in male reproduction is limited due to technical challenges in glycoproteomics.
- This analysis summarizes the current knowledge of glycosylation in the male reproductive system and potential applications of advanced glycoproteomics in male reproduction.
- This analysis presents a comprehensive overview of glycoproteomics in male reproductive diseases.

## Graphical abstract

Glycans and glycoproteins play vital roles in male reproduction. However, the understanding of glycosylation in the physiological and pathological processes of male reproduction is limited due to technical challenges in glycoproteomics. This review summarizes the glycoproteome of human semen, current knowledge of glycosylation in the male reproductive system and associated diseases, progress in glycoproteomic approaches, and potential applications of advanced glycoproteomics in male reproduction.

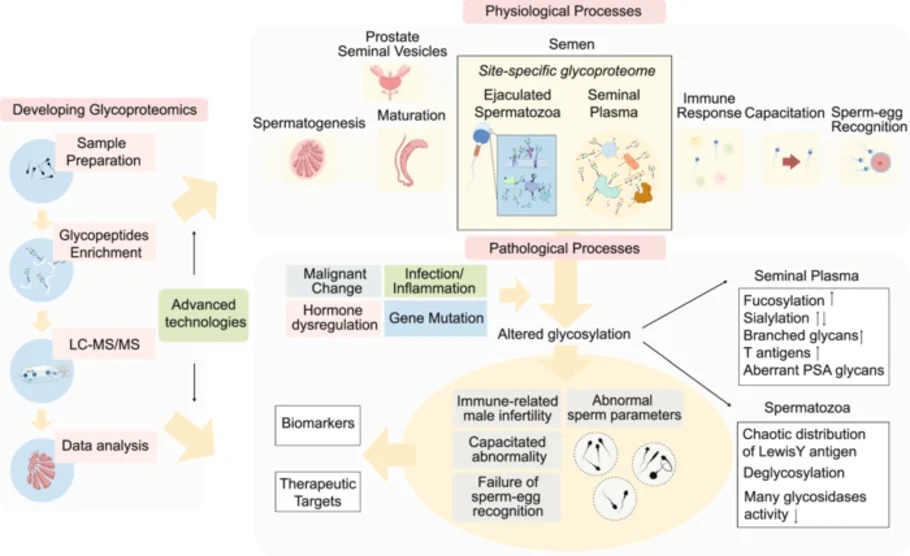

## 1 INTRODUCTION

Glycosylation contributes significantly to male reproduction and is immensely complex, dynamic, active, and regulated throughout spermatogenesis, maturation, mixing with seminal plasma, and entry into the female reproductive tract until fertilization of an egg [1]. A thick glycocalyx‐coated surface consisting of thousands of glycans and glycoproteins, is a remarkable characteristic of human spermatozoa [2]. Seminal plasma, a human body fluid, is highly enriched in such carbohydrate substances [3]. A variety of human spermatozoa and seminal plasma glycoproteins have been identified in the last few decades, and functional characterization has demonstrated that they play a role, particularly in sperm maturation, biophysical changes, immunomodulatory effects, and sperm‐egg binding in the female genital tract [4]. An aberration in glycosylation was found in men suffering from male reproductive disorders such as infertility and prostate cancer, indicating that glycosylation is profoundly involved in the physiological and pathological processes of the male reproductive system [5].

As one of the most abundant posttranslational modifications in organisms, glycosylation is an enzymatic biological process that assembles and modifies glycans on lipids or proteins to synthesize glycolipids and glycoproteins that have various essential functions, such as ligand‐receptor interactions, cellular differentiation, and signal transduction [6]. In addition, it should be noted that proteins can be glycated by nonenzymatic reactions, resulting in the formation of possible harmful advanced glycation end products, which have been observed in the male reproductive system [7]. According to the method of sugar‐protein linkage, protein glycosylation can be divided into two main types: *N*‐glycosylation and *O*‐glycosylation. *N*‐glycosylation, one of the most common and widespread types of protein glycosylation, involving the covalent attachment of glycans to asparagine residues, is categorized into three types: high‐mannose‐, complex‐, and hybrid‐*N*‐glycans [8]. *O*‐glycosylation is also abundant and highly complex in structure, formed via different glycans linked to the hydroxyl groups of serine, threonine or tyrosine, thereby yielding *O‐N‐*acetyl‐galactosamine (*O‐*GalNAc), *O‐N*‐acetylglucosamine (*O‐*GlcNAc), *O*‐galactose, *O*‐mannose, *O*‐fucose, and so forth [9]. *N*‐glycosylation and many types of *O*‐glycosylation occur at the surface of the endoplasmic reticulum (ER), followed by entry into the ER and Golgi apparatus for subsequent processing and elongation. There are some exceptions in which *O‐*GlcNAc was reported in the cytoplasm and nucleus, while *O‐*GalNAc was reported in the early Golgi apparatus [10]. Glycosylation is so widespread that at least half of proteins and most secretory proteins are glycosylated in humans and is a highly complex and step‐by‐step process orchestrated by more than 200 glycosyltransferases and glycosidases through 16 distinct glycosylation pathways rather than the production of nuclear acid or proteins relying on templates [11, 12]. Notably, glycosylation probably holds particular significance for male reproduction. It occurs not only frequently and actively during spermatogenesis but also during sperm maturation, in which process spermatozoa still undergo profound physiological changes that are highly determined by posttranslational modification due to the loss of ability to synthesize proteins de novo, thus necessitating in‐depth investigation [5].

To date, the structure and function of glycosylation in male reproduction remain unclear. Although it is extremely complex and plays a cumulative role, most studies have focused on a type of glycan or a single glycoprotein in male germ cells for a long period of time. Conventional methods to probe glycosylation depend on the specific binding between glycans and proteins, such as antibodies or lectins. In recent years, with considerable progress in omics techniques, the compositions, and structures of a variety of glycans, as well as the glycosites on glycoproteins in the male reproductive system, have been identified and functionally characterized. The glycomes of human spermatozoa and seminal plasma were initially mapped in 2007 and 2009, respectively [13, 14]. A few years later, large‐scale *N*‐glycoproteomes of human spermatozoa and seminal plasma were gradually reported [15, 16, 17]. However, these studies cannot provide detailed glycan structures and their exact glycosites in glycoproteins, which leads to unclear structure‐function correlations. Recently, this drawback has been overcome by taking advantage of large‐scale and site‐specific glycoproteomic approaches [18, 19]. Based on these developments, it can be argued that the *N*‐glycoproteomes of spermatozoa and seminal plasma in men with normal sperm quality have been generally profiled, which lays a solid foundation for further investigation of their associations with male reproductive diseases. Moreover, glycoproteomics is constantly developing and has the potential to refine the male reproductive glycoproteome, identify complex *O*‐glycosylation and address major male reproductive challenges, which are considered among the most severe global public health problems. Considering that a large number of glycoproteins in spermatozoa have been reported to be indispensable for male reproduction and were subjected to exhaustive review, we updated the glycan and glycoprotein profiles of the male reproductive system and its relationship with male reproductive disorders from an omics perspective [2, 4] We also summarize the glycoproteomic techniques applied in male reproduction and the latest progress. This analysis aimed to provide insight into what valuable data and advanced technologies can be used for promoting male reproductive health.

## 2 HUMAN SEMEN GLYCOME AND GLYCOPROTEOME PROFILES

Although intensive research has been conducted on the proteome of the male reproductive tract in recent years, research on the corresponding glycoproteome is still in the initial stages. Earlier studies regarding the glycosylation patterns and glycoproteins of human spermatozoa and seminal plasma have indicated changes in glycosylation throughout the process of spermatogenesis, maturation, and fertilization [3, 20]. Nevertheless, these studies focused on several glycoproteins or glycan patterns and lacked site‐specific information for each glycoprotein, which is far from sufficient to further investigate their functions in detail, such as how each glycan structure on a specific glycosite impacts the function of a specific glycoprotein. Recently, significant progress has been made in understanding the semen glycoproteome, allowing site‐specific *N*‐glycosylation identification and functional characterization of large‐scale glycoproteins, which has laid a foundation for further in‐depth studies on the roles of these glycoproteins in the physiological and pathological processes of male reproduction (Table 1).

**Table 1:** Studies of human spermatozoa and seminal plasma glycomics and glycoproteomics.

Samples	Main methods	Targets	Number of glycosites	Main glycan identifications	Number of glycoproteins	Main functional characteristics	References
Spermatozoa							
Fourteen patients with normal parameters	Swim‐up; MALDI‐TOF MS; lectin and antibody binding	Released *N*‐glycans	/	(1) High mannose; (2) biantennary bisected; (3) heavy fucosylated complex type terminated with Lewis sequences	/	Inhibition of both innate and adaptive immune responses	[14]
Normal healthy adult males	DGC; glyco‐FASP; tandem MS; in vitro fertilization assay	Released *N*‐glycans/deglycosylated glycoproteins	554	(1) More than 90% glycoproteins located in membrane, extracellular region, or lysosome; (2) identify lectin‐like glycoproteins	297	Cell recognition and fertilization	[17]
Ten healthy donors	WHO5; intact glycopeptide approaches and StrucGP software analysis	Precision *N*‐glycan structure at each glycosite	1489	(1) 292 glycan compositions with 719 structures; 1–10 glycosites each protein; (2) high mannose (36.3%) and complex glycans (29.2%) combination of three different types of glycans (11.5%); (3) 4 core and 13 branched structures	968	Spermatogenesis, capacitation, sperm‐egg recognition in uniquely glycoproteins Sialylation/Lewis epitopes in immune response	[18]
Twenty healthy donors	Intact glycopeptide approaches	Site‐specific *O*‐glycoproteins	202	(1) 50% of *O*‐glycoproteins contain one *O*‐glycosite, and the other part contained more than one; (2) most *O*‐glycosites contain one *O*‐glycan type; (3) 105 *O*‐glycosites from 25 *O*‐glycoproteins were found for the first time	68	Multiple biological functions including extracellular matrix structural constituent and binding, which is different from *N*‐glycoproteins	[21]
Seminal plasma							
Healthy donors	Removal of proteins form semen; GC‐MS and MS/MS	Released glycans	/	(1) Fucosylated forms of the disaccharide are major components; (2) glycans rich in fucose and carry Lewis epitopes	/	Fucosylated glycans may promote fertilization	[3]
Four fertile men	Liquefication; centrifugation 1000*g*; MALDI‐TOF MS and MS/MS	Released *N*‐and *O*‐glycans	/	(1) High mannose glycans; (2) bi‐, tri‐, tetraantennary core‐fucosylated complex type *N*‐glycans with antennae terminated with Lewis sequences; and capped with sialic acid; (3) fucosylated/sialylated core 1/2 types	/	Modulation of adaptive but not the innate immune response	[13]
Five healthy adult males (each sample performed separately)	Centrifugation (2500 rpm); LC‐MS/MS; Western blot	Released *N*‐glycans/deglycosylated glycoproteins	720	(1) Similar compositions of glycoproteins among five individuals; (2) 1–10 glycosites each protein	372	Biological adhesion, extracellular matrix organization, glycosaminoglycan metabolic process	[16]
Eighteen fertile men; 72 infertile men	Mixed with buffer and centrifuged at 400*g*; MALDI‐TOF/TOF tandem MS	Released glycans	/	(1) 86 oligosaccharides; (2) high mannose and hybrid type glycans; (3) low terminal sialic acid	/	Altered glycosylation are similar between seminal plasma and sperm surface, disturbing sperm interaction with female immune system	[22]
Seven men with normal semen parameters (pooled)	Intact glycopeptide approaches	Precision glycan structure at each glycosite	73	(1) Complex type (83%), high‐mannose (10%) hybrid (7%); (2) most of glycoproteins are sialylated or fucosylated; (3) many Lewis epitopes bearing glycans were found	50	Immune‐modulating roles of glycoproteins	[15]
Six healthy donors	WHO5; liquefaction; intact glycopeptide approaches and StrucGP analysis	Precision glycan structure at each glycosite	1019	(1) 317 glycan compositions with 773 structures; 1–11 glycosites each protein; (2) complex glycans (45%), high mannose (18%) and combination of three different types of glycans (15%); (3) 4 core and 13 branched structures	620	The majority of identified glycoproteins functioned in response to stimulus and immunity	[19]

Abbreviations: DGC, density gradient centrifugation; GC‐MS, gas chromatography‐mass spectrometry; GlycoFASP, glyco‐filter‐aided sample preparation; LC‐MS/MS, liquid chromatographic tandem mass spectrometry; MALDI‐TOF MS, matrix‐assisted laser desorption ionization time‐of‐flight mass spectrometry; MS, mass spectrometry; MS/MS, tandem mass spectrometry.

### Spermatozoa *N*‐glycome and *N*‐glycoproteome profiles

To date, 1036 *N*‐glycoproteins, which consist of 1489 glycosites, 719 distinct *N*‐linked glycan structures and 292 glycan compositions, have been identified in human spermatozoa. The majority of glycosites were occupied by high mannose‐type *N*‐glycans (36%), complex‐type *N*‐glycans (29%), and a combination of three different types of *N*‐glycans (12%). More than half of the glycoproteins contained only 1 glycosite, 40% of the glycoproteins contained 2–10 sites, and less than 1% of the glycoproteins contained >10 sites [17, 18]. A variety of glycan structures are present in human spermatozoa. The high content of complex glycans suggested their vital roles in spermatozoa, such as spermatogenesis [23]. Compared with earlier studies of glycomics of human spermatozoa, the identification of glycan structures resulted in similar features: high mannose‐type *N*‐glycans, biantennary bisected glycans and heavily fucosylated complex‐type *N*‐glycans terminated with Lewis^x^ and/or Lewis^y^ sequences [14]. Analysis of spermatozoa glycomics revealed 4 types of core structures and 13 types of branch structures. In addition, many glycan compositions consisted of different isoforms (up to 10 isoforms were distinguished). More glycoproteins and details will be continuously identified to improve the understanding of the whole glycoproteome. The functions and pathways of the identified glycoproteins were further explored by gene ontology (GO) enrichment analysis but remain unclear. Generally, these genes were involved in immune functions and sperm‐egg interactions. Notably, compared with the proteome, the human spermatozoa glycoproteome contains the specific glycoproteins, while proteomic analysis of human spermatozoa has demonstrated that their major function is metabolism [24].

The glycoproteomic analysis of human sperm revealed several distinctive features. Most significantly, heavy fucosylation (>50%) was detecyed in human spermatozoa, and up to 10 fucoses per glycan were detected on some glycosites. Clusterin has been found to be highly fucosylated, suggesting its roles in immune tolerance and capacitation in the female reproductive tract [18]. Second, human sperm contain *N*‐glycans of the biantennary bisecting type and oligosaccharides terminated with Lewis antigens pertaining to the suppression of both natural and adaptive responses [14]. Additionally, more than 200 glycoproteins have been identified exclusively in spermatozoa and are not found in human tissues or cells. Functional enrichment analysis demonstrated that these glycoproteins were involved in very specific spermatozoa‐related biological processes, such as sperm‐zona pellucida binding, spermatogenesis, sperm‐egg recognition, and fertilization. Pathway analysis revealed acrosome reaction, sperm‐oocyte membrane binding and sperm motility [18]. A previous report revealed that *N*‐glycoproteins in spermatozoa are mainly involved in cell recognition and fertilization, particularly gamete interactions. The glycosaminoglycan degradation and lysosome pathways are highlighted [17]. In addition to glycan compositions and structures in spermatozoa, some specific glycosylation features in different locations of human spermatozoa, including total spermatozoa, plasma membrane, extracellular region, acrosome, ER, lysosome, and Golgi apparatus, were identified. More than 90% of sperm glycoproteins are located in the membrane, extracellular region, and lysosome [17]. Special attention was given to the acrosome region, in which glycans may participate in capacitation and sperm‐egg recognition. The bisected core structure (with and without core fucosylation) was more highly expressed in the acrosome than in the glycoproteins at other locations and in total spermatozoa. Many glycoproteins in the acrosome of sperm, such as acrosin, may be derived from lysosomes, such as the lysosomal membrane *N*‐glycoprotein 1 (LAMP1) and 2 (LAMP2) in the sperm glycoproteome. However, a more precise location of the glycan structure needs to be clarified. In addition, the number of special branch structures of the GalNAcβ1‐4GlcNAc motif (also known as *N,N'*‐diacetyllactosediamine, LacdiNAc) was greater in acrosomes than in total spermatozoa. LacdiNAc structures and bisected core structures may contribute to special functions of the acrosome. The glycoproteome of spermatozoa also revealed information on sperm development. Twenty‐seven testis‐specific glycoproteins were identified in mature spermatozoa. Compared with the proteome of human epididymal spermatozoa, 14 overlapping genes were identified, in agreement with the critical role of the epididymis in sperm glycocalyx modification [25]. In addition, 114 genes of the sperm glycoproteome were also detected in the seminal fluid [26]. Although sperm contact seminal plasma constructed from the prostate and seminal vesicle for a short duration, they may still attach to the sperm surface and be important for male fertility, which was proven by the substitution of foreign healthy seminal plasma to improve sperm parameters [27]. However, their interaction needs to be further elucidated.

### Seminal plasma *N*‐glycome and *N*‐glycoproteome profiles

The *N*‐glycoproteome map of human seminal plasma was established in recent years by several studies, with 816 glycoproteins comprising 773 *N*‐glycan structures, 317 *N*‐glycan compositions and 1019 *N*‐glycosites. The majority of glycosites were occupied by complex‐type *N*‐glycans (45%), followed by high mannose‐type *N*‐glycans (18%) and a combination of hybrid‐type and complex‐type *N*‐glycans (15%) [15, 18]. Approximately 70% of glycoproteins contained only one glycosite, glycoproteins containing more than 5 glycosites accounted for 5%, and a maximal of 11 glycosites were found in attractin, which were proven to be associated with male reproduction [28]. The main glycan structures of seminal plasma are similar to those of glycans in spermatozoa. The presence of complex‐type glycans is indispensable for glycoprotein secretion, and high mannose‐type glycans participate in immunomodulatory interactions [22]. Similarly, the functions and pathways of these glycoproteins in seminal plasma were explored using bioinformatic analysis. The results of the functional enrichment analysis differed among several studies, which was due to the different numbers of glycoproteins identified. For example, the most significantly enriched term in biological processes (BP) was biological adhesion, followed by extracellular matrix organization [16]. In summary, it can be concluded that the majority of identified glycoproteins function in response to stimuli and in immunity, adhesion, motility, and chemotaxis. Compared with functional enrichment analysis of the proteome of human seminal plasma, there was a significant difference [24].

Despite the high intersubjective variability of seminal plasma glycomics, heavy fucosylation (fucose residues ≥6 per glycan) was found in seminal plasma glycoproteins, similar to human spermatozoa. The presence of up to nine fucose residues in one glycan was detected in seminal plasma from healthy men. Core and antennary fucosylation have been reported for the majority of seminal plasma glycoproteins, such as fibronectin, glycodelin S and prostate‐specific antigen (PSA). The interactions of sperm with the antennary of fucose residues, especially those of the Lewis^x^ type, are more important than those with proteins located in the glycan core [29]. The heavy fucosylation of clusterin and galectin‐3‐binding protein indicated that these glycoproteins may be involved in the immune response. However, functional enrichment analysis of glycoproteins without heavy fucosylation also revealed biological processes of the immune response, which demonstrated the complexity and heterogeneity of glycoproteins in seminal plasma. A recent study showed that 60% of glycan structures in seminal plasma were sialylated [19], which is different from a previous report of approximately 30% [22]. Sialic acid and fucose may exert synergistic effects on the immune response. Notably, approximately half of glycoproteins are secreted proteins [16]. However, the source of the glycans and glycoproteins in seminal plasma from accessory sexual glands has received much less attention. By comparing the glycoproteome with protein biomarkers in human seminal plasma, 11 glycoproteins were identified as epididymis‐derived glycoproteins, and 10, 4, and 3 glycoproteins were identified as testis‐, seminal vesicle‐, and prostate‐derived glycoproteins, respectively [30]. The complete and more accurate glycoproteome of accessory sexual glands remains to be established.

### Recently‐mapped human semen *O*‐glycoproteome

Compared with that of *N*‐glycosylation, the high complexity of *O*‐glycosylation poses extraordinary technological challenges [9]. Although *O*‐glycans and *O*‐glycoproteins of human semen have been explored in recent years, the first *O*‐glycoproteome map with site‐specific information was generated by our group using a novel strategy named glycoproteomics based on two complementary fragmentation methods (GlycoTCFM) [21]. We found 68 *O*‐glycoproteins containing 371 intact *O*‐glycopeptides and 202 *O*‐glycosites in total, among which 30 and 25 were specifically identified in spermatozoa and seminal plasma, respectively. Unlike *N*‐glycoproteins, 13 *O*‐glycoproteins overlapped. Thirty‐four *O*‐glycoproteins possessed one *O*‐glycosite, and the other possessed two or more *O*‐glycosites. For *O*‐glycans, 141 *O*‐glycosites contained one, and 61 contained more than one. Functional enrichment analysis revealed the role of *O*‐glycoproteins in cell adhesion and angiogenesis. Notably, two abundant and complex *O*‐glycoproteins (semenogelin‐1 and semenogelin‐2) produced by the seminal vesicle and one (equatorin) located in the acrosome membrane of sperm, were highly *O*‐glycosylated, with more than 20 *O*‐glycosites. The established *O*‐glycoproteome of human semen could lay a foundation for the development of *O*‐glycoproteomic technology and knowledge of *O*‐glycosylation in male fertility and infertility.

### Commonality and uniqueness of spermatozoa and seminal plasma glycome and glycoproteome profiles

**Figure 1:**
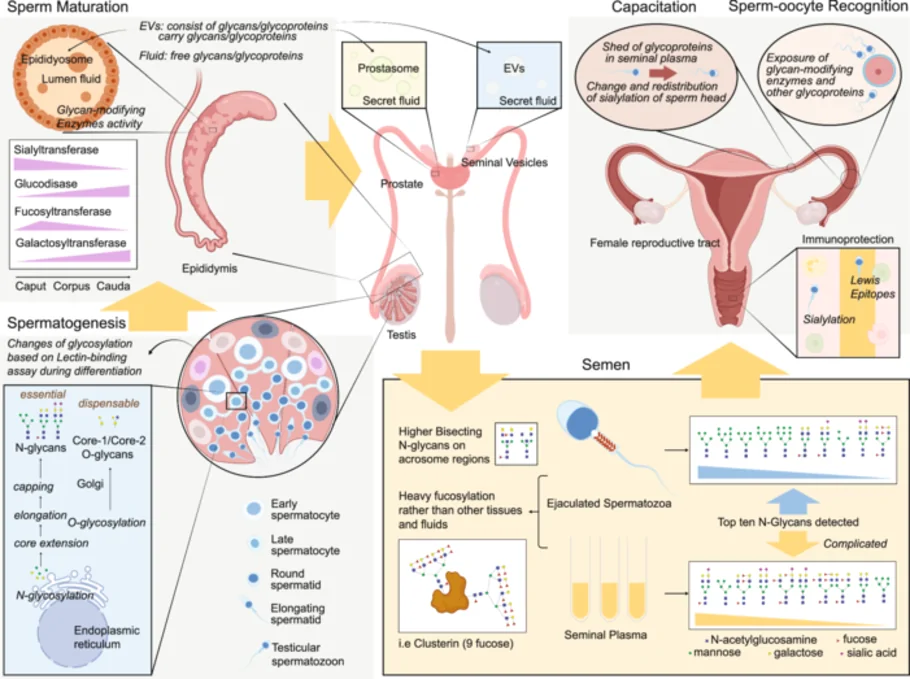
An overview of highly active, complex, spatiotemporal, and regulated glycosylation throughout the entire lifespan of human spermatozoa. During spermatogenesis, both N‐glycans and O‐glycans are actively synthesized. There were marked changes in glycosylation during each period of sperm differentiation. Glycosylation continues during sperm maturation and is orchestrated by various glycan‐modifying enzymes. In addition, sperm acquire glycoproteins delivered by epididymosomes. Ejaculated sperm can capture abundant extracellular vesicles (EVs) from seminal plasma, which contain glycoproteins secreted by the prostate and the seminal vesicle. The ejaculated sperm and seminal plasma glycoproteome were profiled. Both of these proteins function in spermatozoa survival in the female genital tract and sperm‐egg recognition (figure was created with Biorender.com).

The glycoproteomics of spermatozoa and seminal plasma were often conducted by the same group and then compared with each other. Generally, the composition of glycoproteins highly overlaps. First, glycomic analysis revealed substantial similarity, including abundant glycans terminated with Lewis^x^ and/or Lewis^y^ sequences. W, bisecting‐type *N*‐glycans are significantly less abundant in seminal plasma than in sperm. The levels of fucosylated and sialylated glycans in both sperm and seminal plasma differed dramatically among individuals [13]. Further glycoproteomic analysis revealed that 383 glycoproteins and 499 glycan structures overlapped in human seminal plasma and spermatozoa [19]. Nevertheless, approximately half of the glycan structures were identified exclusively either in spermatozoa or in seminal plasma. Even the glycoproteins shared between them varied in their specific structure. Compared with spermatozoa, seminal plasma possesses more complicated glycan structures. Moreover, fucosylated core structures were predominant in seminal plasma rather than common core structures in spermatozoa. Consistent with the glycomic results, the expression of bisecting core structures in seminal plasma was greatly decreased compared to that in spermatozoa. However, complicated fucosylated and sialylated branch structures were more common in seminal plasma. In contrast, simple branch structures, such as high mannose and LacdiNAc. The above differences may be biologically significant (Figure 1).

The identification of the human spermatozoa glycoproteome revealed many distinctive characteristics compared with those of other cell types in the body. Notably, no heavy fucosylation was detected in other human tissues except for spermatozoa. For instance, a maximum of 4 fucoses per glycan were identified on clusterin in the liver, and no fucose was identified on clusterin in mouse tissues [31]. Interestingly, altered fucosylation was detected in cancerous cells and regenerative tissues. For example, prostate cancer cells express elevated levels of fucosylated glycoproteins, probably due to changes in fucosyltransferase and fucosidase levels [32]. In the neonatal mouse heart, increased core fucosylation was associated with a decrease in regenerative capacity [33]. These unique features of the spermatozoa glycoproteome and its relationship with or without proliferative ability need to be investigated.

Similarly, the identification of the seminal plasma glycoproteome revealed many distinctive characteristics compared with those of other body fluids. Unlike seminal plasma, no heavy fucosylation was found in other body fluids. For instance, there is only one fucose per glycan on clusterin in serum. In addition, one of the most unusual aspects of glycosylation in humans is the very high expression of terminal Lewis^x^/Lewis^y^‐type sequences associated with all the main types of glycoconjugates. In particular, Lewis^y^ sequences are almost not detected in other human cell types or secretory products [13]. In human serum, half of the *N*‐glycosites are modified by at least two glycans, which is more than what has been observed in seminal plasma. Moreover, sialylation in serum is greater than that in seminal plasma [34]. In human urine, most glycopeptides are complex or hybrid glycans composed of sialic acid and fucose, and these glycopeptides are reported to be similar to those in serum [35]. Surprisingly, approximately 800 glycoproteins were identified in seminal plasma, more than in serum and urine, although this information was obtained from the whole body. The reported glycoproteome of seminal plasma indicated its abundance and reflected the unique source pertaining to a specific function.

### Glycome and glycoproteome profiles during spermatogenesis, sperm maturation, and capacitation

The development of human sperm, maturation and capacitation are all concomitant with continuous changes in glycosylation (Figure 1). Scientists have studied this phenomenon for many years and have attempted to determine glycosylation profiles spatiotemporally during this process. The glycosylation pattern of human intratesticular spermatogenic cells and ejaculated spermatozoa was first established by classical lectin binding assays. During differentiation, spermatogenic cells are specifically reactive to lectins. Ejaculated spermatozoa showed reactivity with all 20 lectins but could be further grouped according to the binding sites of the head region [20]. Similarly, lectin binding‐based strategies have been applied to testicular, epididymal and ejaculated sperm from mice, cats, and dogs [36].

Lectin‐based assays indicated marked changes in glycan content during spermatogenesis. Further studies revealed that complex *N*‐glycans, rather than hybrid or oligomannose *N*‐glycans on glycoproteins, were required for sperm development. Neither core 1 nor core 2 O‐GalNAc glycans nor *O*‐fucose glycans are essential for this process [23, 37]. Compared with those during spermatogenesis in the testis, alterations in glycomic profiles during sperm maturation and capacitation have received more attention, and studies have focused on the sialylation of glycoproteins. Many earlier studies revealed alterations in glycosylation patterns in the epididymis of many mammals by investigating surface charge or lectin‐binding features [38]. Peptide *N*‐glycosidase F (PNGase F) and liquid chromatographic tandem mass spectrometry (LC‐MS/MS) were used to explore changes in *N*‐linked sialylated glycopeptides during epididymal transit in rats. Ninety‐two unique *N*‐linked sialylated glycopeptides were identified from 65 different glycoproteins, and maturation‐related remodeling of the *N*‐linked sialylated glycoproteins was detected in the caput, corpus and cauda spermatozoa [39]. For sperm capacitation and the acrosome reaction, sialylated monosaccharides were found to be essential. Some sialylated monosaccharides are shed from the sperm surface during capacitation and are controlled by sialidases. The decreased expression of these genes may be related to idiopathic infertility in some males [40]. Similarly, studies using boar spermatozoa detected changes in the distribution of *N*‐acetylglucosamine, sialic acid, mannose, and fucose residues during capacitation and the acrosome reaction [41]. A more precise glycoproteome of spermatogenesis, maturation, and capacitation requires further investigation.

### Profiles of enzymes participating in the formation and modification of the glycome and glycoproteome in male reproduction

Glycosylation continues throughout spermatogenesis, maturation, and capacitation and is indispensable for developing functionally competent spermatozoa to fertilize an egg. Fulfillment of the above processes results from a cumulative effect of various glycan‐modifying enzymes present in the male reproductive system, which are also stored in spermatozoa and participate in fertilization directly. Glycan‐modifying enzymes show distinct expression patterns during different periods of sperm production, suggesting that they play spatiotemporally specific regulatory roles. For instance, *β*‐*D*‐galactosidase was found to vary in spermatocytes, round spermatids, caput, and cauda spermatozoa [42, 43]. In addition, fucosidase and mannosidases were detected in testicular spermatozoa and the sperm plasma membrane. Although these enzymes play dispensable roles in male reproduction, a few studies have revealed their quantity and function, some of which are listed in Table 2.

**Table 2:** Glycan‐modifying enzymes involved in male reproduction.

Species	Name	Biological process	Location	Function	Outcome	References
Mice	*α*1,2‐fucosyltransferase	Spermatogenesis	Testis	Fucosylation of glycolipids during spermatogenesis	It needs either the *FUT1* or *FUT2* gene	[44]
Mice	UDP‐galactose: Ceramide galactosyltransferase	Spermatogenesis	Testis	Precluding the addition of Gal to the precursor of seminolipid	No spermatids or sperm	[45]
Mice	*α*‐mannosidase II (Man2a2)	Spermatogenesis	Testis	*N*‐glycans synthesis	Infertile, smaller testis, fewer spermatids, and immature sperm	[46]
Mice	GalNAc transferase (Galnt3)	Spermatogenesis	Testis	Synthesis of *O*‐GalNAc glycans in the Golgi	Disrupt spermatogenesis and male infertility	[47]
Mice	GlcNAc transferase (Lfng)	Spermatogenesis	Golgi	Defective development of nongerm cells	Defective rete testis	(48]
Mice	*β‐D*‐galactosidase	Sperm maturation	Epididymis luminal fluid	Modification of terminal galactosyl moieties on the sperm surface	Gradual decrease through transition from the distal caput to the proximal cauda epididymis	(49]
Mice	Fucosyltransferase; sialyltransferase	Sperm maturation	Epididymis and spermatozoa	Binding with the endogenous sugar acceptor site on the sperm surface	Gradual decrease in the spermatozoa through transition from the distal caput to the proximal cauda epididymis	[50]
Mice	*β*1,4‐galactosyltransferase	Fertilization	Spermatozoa	Capacitation and taking part in sperm‐egg interaction; mediating sperm‐egg interaction	Overexpressing of it in sperm result in unable to bind eggs	[51]
Man	*O*‐GalNAc‐transferase (GalNAc‐T3)	Fertilization	Spermatozoa	Abnormal maturation and reduced functionality	Significantly lower in OAT patients	[52]
Man	*α*‐galactosidase; *β*‐galactosidase; *α*‐glycosidase; *β‐N*‐acetylglucosaminidase	Fertilization	Spermatozoa	Interfering with the interaction between gametes	Significantly lower in OAT patients than fertile controls	[53]
Man	*α*‐mannosidase	Fertilization	Seminal plasma	Effect the penetration of human spermatozoa through the zona pellucida	Significantly higher in OAT patients than fertile controls	[53]
Man	Fucosyltransferase	Fertilization	Seminal plasma	Interfering and disturbing the normal course of the fertilization cascade	Subfertility	[29]
Man	Fucosyltransferase 3 and 4	Fertilization	Seminal plasma	Causing clusterin fucosylation changes among male fertility disorders	Association with male infertility	[54]

Abbreviations: Gal, galactose; GalNAc, N‐acetylgalactosamine; GlcNAc, N‐acetylglucosamine; OAT, oligoteratoasthenozoospermia; UDP, uridine diphosphate.

Glycan‐modifying enzymes involved in male reproduction can be divided into two main types, glycosyltransferases (synthetic) and glycosidases (hydrolytic), which work jointly to form and modify the sperm glycocalyx. Most glycan‐modifying enzymes are also *N*‐glycoproteins, which contain multiple glycan chains of high mannose, complex, and perhaps hybrid types [55].

During spermatogenesis, glycan‐modifying enzymes responsible for the synthesis of glycans, glycoproteins, and glycolipids mainly reside in the endoplasmic reticulum and Golgi apparatus of immature sperm cells. These enzymes include more than 100 types and exhibit distinct expression patterns during different periods and at different locations. For example, *α*‐mannosidase II is an enzyme involved in *N*‐glycan synthesis that is enriched in all germ cells except spermatogonia and condensing spermatids. Deletion of this gene in mice results in infertility, resulting in smaller testes and immature sperm, such as multinuclear cells [46]. GalNAc transferase is expressed in the Golgi of spermatids and spermatocytes to synthesize *O‐*GalNAc glycans. Ablation of this gene decreases acrosome formation and increases apoptosis during spermatogenesis [47]. The *β*−1,4‐galactosyltransferase family performs *β*−1,4‐galactosylation in the process of glycosylation. The expression of these genes is altered significantly at different stages of testis development and spermatogenic differentiation, indicating differential galactosylation of testis glycoproteins [56]. In addition, the fucosylation of glycolipids in testes was associated with spermatogenesis via the use of *α*1,2‐fucosyltransferase, which requires the expression of either the *FUT1* or *FUT2* gene [44].

When sperm exit the seminiferous tubules and transit through the epididymis, glycan‐modifying enzymes in the epididymal lumen fluid predominate sperm glycosylation, making use of free sugar donor substrates and extracellular vesicles (EVs) carrying glycans [50, 55]. For example, *β*‐D‐galactosidase modifies terminal galactosyl moieties on the surface of sperm [49]. In addition, glycan‐modifying enzymes such as fucosyltransferase and sialyltransferase in the epididymal lumen fluid can bind with sperm during maturation. For example, activities were greater in the caput region of the rat epididymis than in the cauda region (Figure 1). However, sialylation clearly occurs during sperm transit.

Many glycosidases, also known as glycohydrolases, such as galactosidase, fucosidase, mannosidase, and glucosaminidase, are enriched in seminal plasma and show high specificity [43, 57, 58]. Significantly higher fucosyltransferase III activity was detected in human seminal plasma, which is consistent with high fucosylation [59]. According to the glycoproteome of human seminal plasma, 10 glycosyltransferases and 10 glycosidases were identified [19]. Together, the above enzymes constitute a highly specific, complex, and dynamic enzyme profile in the male reproductive system, and our understanding of this profile is far from complete. The glycan‐modifying enzyme profile is also a significant part of the glycoproteome of male reproduction.

### Emerging vital functions of EVs in semen glycosylation

Posttesticular glycosylation of spermatozoa is regulated by extracellular glycan‐modifying enzymes in the epididymal fluid, as mentioned above. In addition, male reproductive tract EVs are strongly implicated in this process [60]. EVs are small membrane‐bound particles that encapsulate various molecules. They are highly abundant and diverse in seminal plasma and are involved in each period of sperm development, whereas their roles in post testicular modification have attracted major interest [61]. Seminal EVs originate from the epididymis (epididymosomes), vas deferens, and other accessory sexual glands, especially the prostate (prostasomes). These glands exert their functions by releasing EVs into the male genital tract. Subsequently, EVs fuse with the sperm membrane, sending substances such as nucleic acids, lipids, proteins, and glycans that are required for sperm maturation or remaining in the fluid, mediating the contact of sperm with the complex milieu in the female reproductive tract [62] (Figure 1).

EVs contain various proteins, convey specific glycan epitopes and carry glycans either on their surface or as cargo [63, 64]. Since human sperm lose the ability to synthesize glycans and glycoproteins de novo after exiting the seminiferous tubules, posttesticular glycosylation is highly mediated by EVs. It is recognized that epididymosomes, membranous vesicles secreted by epididymal epithelial cells, transfer proteins into specific regions of sperm during epididymal transit. Many of these proteins are well‐known glycoproteins that influence male fertility, such as P25b, which is involved in zona pellucida binding; PH‐20, which relocates during sperm maturation; and HE5/CD52, which is expressed in the distal corpus and cauda regions [65, 66]. CD44 may be a possible marker of epididymosomes in boar ejaculate [67]. Therefore, aberrant expression of glycoproteins in EVs may contribute to male infertility. A proteomic study of seminal plasma exosomes revealed that glycodelin was overrepresented in asthenozoospermic men, resulting in a normal capacitation process [68]. In addition to glycoproteins coated in EVs, the glycan patterns of seminal EVs may participate in interactions between them and immune cells in the reproductive tract [69, 70]. Common *N*‐ and *O*‐glycosylated species were found in EVs extracted from the seminal plasma of healthy men. Moreover, EVs showed glycosylation signatures distinct from those of the supernatant, including increased levels of sia‐*α*−2,6‐*Gal* and *N*‐glycans and increased affinity of dendritic cell‐specific intercellular adhesion molecule‐3‐grabbing non‐integrin (DC‐SIGN) and sialic‐acid‐binding immunoglobulin‐like lectin‐9 (Siglec‐9) for their lectin ligands [71]. Abnormal changes in seminal EV glycosylation can occur under different physiological and pathophysiological conditions. For instance, in prostasomes from normozoospermic and oligozoospermic men, surface *N*‐glycans with sialylated and mannosylated moieties, which form part of the vesicle coat, showed slight differences, whereas the redistribution of integral membrane proteins showed greater differences, as revealed by tetraspanin and galectin‐3 assembly. Together, these molecules may establish the glycosylation patterns of the prostasome [72]. Since prostasomes are major contributors to the EV composition in seminal plasma and have beneficial effects on sperm motility, immunosuppressive activity and acrosomal reactivity, their potential as biomarkers should be highlighted [73]. Clearly, glycans and glycoproteins contribute to seminal EV composition and play a significant role in the male reproductive system. However, little is known about their glycoproteome profiles and possible biological functions. Proteomics analysis revealed 1282 proteins in the human prostasome and a total of proteins in the mouse epididymosome, providing a solid foundation for further studies focusing on glycoproteomics [74].

## 3 EFFECTS OF THE SEMEN GLYCOME AND GLYCOPROTEOME ON MALE REPRODUCTIVE HEALTH

Given the indispensable role of glycoproteins in male reproduction, it is reasonable to think that some male reproductive diseases are related to altered glycoproteomes. Although the site‐specific glycoproteome of semen from healthy men has been established recently, precision glycoproteomics has not been applied in men with reproductive disease. Currently, these methods are limited by the presence of a small number of glycoproteins or glycans [22, 75]. Lectin‐based methods cannot accurately reflect the structure of oligosaccharides. It may bind less specific but abundant structures and may also not bind the glycans that are inside the glycoprotein structure. Nevertheless, glycomics or strategies using lectin‐binding assays combined with mass spectrometry (MS) have provided much information in the field of male reproductive health, most of which has focused on seminal plasma. These studies, although not perfect, allowed us to summarize the altered profile of the semen glycome and glycoproteome in terms of pathological status. In the current analysis, we discuss these issues from the perspective of glycomics and glycoproteomics. We concluded that an altered human semen glycoproteome was observed in infertile men and contributed to some cases of male infertility, especially immune‐related and fertility defects, and shed light on unexplained male infertility. Although studies regarding semen glycoproteomics of infertile men remain scarce and have mostly been conducted using seminal plasma, studies focusing on the glycoproteome highlight its role in male infertility and provide clues for its diagnosis and treatment. It should be noted that glycomics may reveal information regarding the immune response, and the combination of glycans and proteins results in more complicated functions; that is, glycomics and glycoproteomics may provide information separately and complementarily.

### Altered semen glycome and glycoproteome and underlying mechanisms leading to male infertility

**Figure 2:**
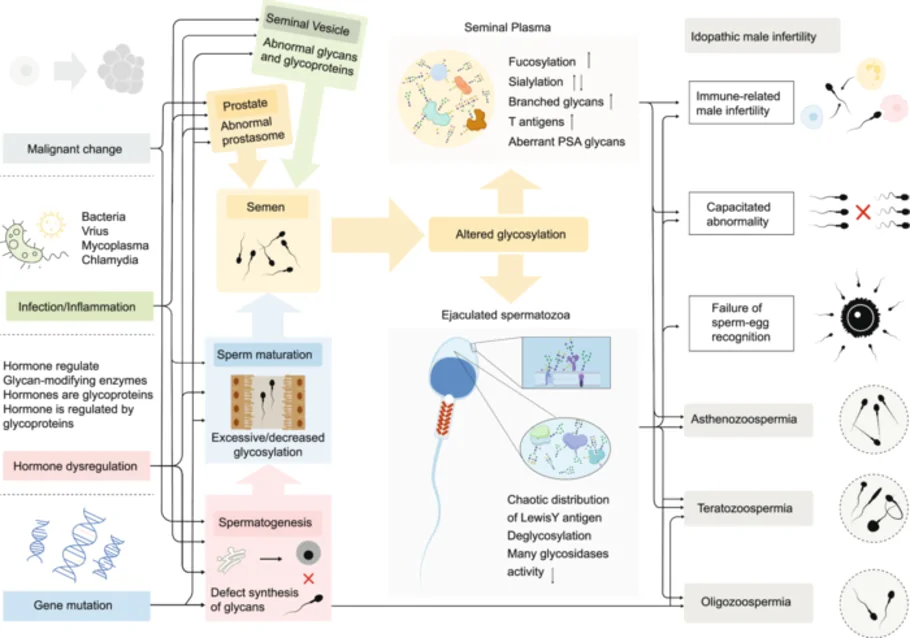
The pathogenesis of altered male reproductive glycosylation associated with male reproductive disorders. Redistribution of glycans, deglycosylation, and decreased glycan‐modifying enzymes in spermatozoa may lead to infertility. In addition, elevated fucosylation and changes in sialylation and specific glycans are found in seminal plasma. The underlying mechanism of the alteration includes gene mutation, hormone dysregulation, infection or inflammation and carcinogenesis, impairing glycosylation during spermatogenesis, maturation, and the function of accessory sexual glands. These factors cumulatively result in asthenozoospermia, oligozoospermia, and sexual gland diseases such as prostate cancer (figure was created with Biorender.com).

The incidence of male infertility has increased in recent years. The etiology of male infertility is complex and largely unknown, which may be attributed to glycosylation abnormalities (Figure 2). The physiological role of glycosylation in human spermatozoa has received increasing attention, but studies focused on aberrant glycosylation patterns are scarce. It was proven that the removal or aberration of glycans on glycoproteins in spermatozoa leads to sterility [17]. Moreover, the defective spermatozoa showed a chaotic distribution of high levels of Lewis^y^ expression on their surface in comparison with normal spermatozoa, which possessed a well‐organized distribution of *N*‐glycans [14]. Furthermore, some lectin‐binding sites were reduced in spermatozoa from oligospermic individuals, suggesting that molecular rearrangement of the sperm membrane occurs during infertility [76]. Notably, compared with studies on spermatozoa, studies regarding the correlation between glycosylation and male infertility have been conducted on seminal plasma. Seminal plasma has long been considered only a cargo and gas station for sperm. In recent years, its role has been reconsidered more important, and it can provide valuable information for the diagnosis and treatment of male infertility and accessory sexual gland dysfunction [77]. The function of seminal plasma largely relies on the abundant and unique glycoproteins it contains. Seminal plasma glycans were found to affect male fertility potential by playing important roles in immune regulation and preventing early capitation of sperm [78]. When spermatozoa enter the female reproductive tract post cotise, they must avoid attack from immune cells in the vagina because they are identified as autografts by the maternal immune system. The underlying mechanism remains unclear; nonetheless, it is generally accepted that seminal plasma glycans play an important role as immunomodulatory substances, according to the well‐known hypothesis of the “human fetoembryonic defense system” [79]. The mechanism of immune tolerance regulated by glycans in seminal plasma is associated with several distinct glycosylation features that are rare in other human body fluids. Seminal plasma unique glycoproteins enriched in immune‐related glycoepitopes that may act as ligands for endogenous lectins exist on the surface of immune cells [80]. These glycans included Lewis^x^ and Lewis^y^ antigens, bisecting GlcNAc, terminal sialylation and high‐mannose type sequences. Recently, some truncated *O*‐glycans, including Thomsen‐Friedenreich (T) and Thomsen‐nouveau (Tn) antigens, as well as their sialylated versions (sT and sTn), have also been shown to be immunomodulatory. In addition to their immunomodulatory role, highly specific glycans in seminal plasma also help maintain the fertility competence of spermatozoa in the maternal environment. A typical example is glycodelin, which plays a crucial role in sperm capacitation, the acrosome reaction and sperm‐oocyte binding [81]. Similarly, alterations in the levels of some glycans in seminal plasma may interact with the sperm surface and disturb normal fertilization processes.

First, early studies focused on some unique glycosylation patterns of seminal plasma in infertile men using lectin recognition of glycoprotein sugar moieties combined with MS [29, 82]. A decrease in the content of *N*‐glycans is presumed to be present in infertile men [83]. As high fucosylation is a significant feature of human seminal plasma, a detailed analysis revealed an even greater content of fucosylated glycopeptides in the seminal plasma of infertile/subfertile men. Fucose, as a Lewis‐type antigen, mediates sperm‐zona pellucida binding, and the addition of fucose‐containing glycans in vitro results in the suppression of their binding [29]. Therefore, the excessive fucosylation of glycans may disturb their mutual interactions and affect male fertility [29]. These fucosylated glycopeptides were further proven to be present in many typical seminal plasma glycoproteins, such as prostate‐specific antigen, *α*‐acid glycoprotein (AGP), glycodelin, and fibronectin. However, this study cannot determine which protein is responsible for the observed increase in fucosylation, which may serve as a potential biomarker in the seminal plasma of infertile males. Indeed, the increase in the total fucose content may be related to either elevated expression of glycoproteins carrying fucose or intensification of the fucosylation pathway itself, independent of protein synthesis but associated with enzyme activity [29]. An example of the independence of glycans from glycoproteins was found in the comparison of PSA glycosylation between fertile and infertile men. As shown in the abovementioned studies, male infertility was concomitant with glycan alterations, while there was no difference in PSA glycosylation between fertile and infertile men [84]. Additionally, glycoproteins may exist as multiple glycoforms in which different isoforms share identical protein but not glycan structures. For example, glycodelin is one of the most important glycoproteins in reproduction; it has 4 glycosylations, but each glycoform has a specific function such that the glycan structures on the proteins determine its function [81]. This finding verifies the involvement of these proteins in fertilization events. Moreover, it has been demonstrated that not only protein expression but also surface glycans may be crucial [75]. In addition to fucosylation, the display of high‐mannose and hybrid‐type oligosaccharides in seminal plasma proteins has also received much attention and was found to be significantly decreased in oligozoospermic patients [82]. Furthermore, 17 glycoproteins were identified, and 1/3 of the oligosaccharides in these glycoproteins were found to express terminal mannose residues. Notably, the levels of high‐mannose and hybrid‐type glycans in normozoospermic infertile patients were also lower than those in fertile men, which may be related to preterm capacitation [83]. In addition, a detailed analysis of terminal sialylation and the expression of Lewis^x^ and Lewis^y^ glycoantigens in the seminal plasma of fertile and infertile men revealed that many glycoproteins carrying these immunomodulatory epitopes were altered, at least in some groups of infertile subjects. These decorations may be essential for carbohydrate‐protein cross‐talk during fertilization [75].

Subsequently, glycomics of fertile controls and infertile patients with normozoospermia, oligozoospermia, asthenozoospermia, and oligoasthenozoospermia were compared using matrix‐assisted laser desorption ionization time‐of‐flight mass spectrometry (MALDI‐TOF‐MS) analysis. Generally, 86 *N*‐glycans were identified in all the analyzed samples, and high mannose and low sialic acid terminations were found in seminal plasma, consistent with previous reports. There was a significant decrease in sialylation and greater fucosylation in the fertile control group than in the infertile group. However, when comparing the fertile control group with the infertile group with respect to normal sperm parameters, highly branched glycans and sialylated oligosaccharides were significantly elevated [22].

Research related to the analysis of seminal plasma glycosylation patterns has focused on glycoepitopes containing fucose and sialic acid. However, such terminal glycoepitopes can exist on both *N*‐ and *O*‐glycans. In the recent years, studies have focused on the *N*‐glycome because of its technical accessibility. This approach, however, ignores an important category of truncated *O*‐glycans, including T and sialo T/Tn antigens, although they are also known to play an immunomodulatory role. An earlier study reported that the expression of *Sambucus nigra agglutinin* (SNA)‐reactive sialic acids significantly differed between the asthenozoospermic and normozoospermic groups [82]. Mucin‐type glycosylation was also analyzed, indicating potential reactivity with DC‐SIGN lectin annotated to *O*‐linked Lewis*‐*type antigens [71]. Moreover, T/Tn antigens and related structures in seminal plasma glycoproteins were investigated recently by using lectins [80]. This study confirmed the predominance of T over Tn antigen for some glycoproteins in infertile subjects compared to the control group. Lactotransferrin, prolactin inducible protein, fibronectin and Semenogelins 1 and 2 were also identified as proteins with glycans terminated with Gal or GalNAc residues. Furthermore, the identified glycoproteins are thought to be involved in immune processes, indicating their possible role in modulating the immune response in the female reproductive tract [80].

### Pathogenesis of the altered semen glycoproteome in the male reproductive system

The above studies confirmed the presence of altered glycomics in the seminal plasma of infertile men. However, the reason behind this alteration remains dismal and is supposed to be a culminative result of male reproductive system dysfunction (Figure 2). Some possible underlying mechanisms leading to the deterioration of the glycoproteome in semen are listed below.

#### Genetic disorders

Unlike mono gene mutations, which cause a single glycoprotein abnormality, mutations in genes encoding glycan‐modifying enzymes affect the entire glycosylation pattern, disrupting the synthesis of glycans and hyper/hypo glycosylation [85]. In recent years, the effect of glycosylation‐related enzymes on spermatogenesis has been determined by the knockout of corresponding genes in mice. The most prominent *N*‐glycan‐modifying enzymes that are necessary for sperm development include the *α*‐mannosidase IIx encoded by *Man2a2*, one of the enzymes that prunes mannose in *N*‐glycans, in accordance with the high occupation of high mannose‐type *N*‐glycan on sperm glycoproteins. However, the depletion of other *α*‐mannosidase II is unaffected [37]. In addition, the GlcNAc transferases MGAT1 (*α*1,3‐mannosyl‐glycoprotein 2‐*β*‐*N*‐acetylglucosaminyltransferase) and MGAT2 (uridine diphosphate, UDP‐*N*‐acetylglucosamine: α−6‐*D*‐mannoside *β*1,2‐*N*‐acetyl‐ glucosaminyltransferase II) are indispensable for spermatogenesis. Compared with enzymes that modify *N*‐glycans, the ablation of *O*‐glycans, such as some glycosyltransferases synthesizing core 1 and 2 *O‐*GalNAc glycans in the Golgi, seemed to be less important. However, another GalNAc transferase family, named GALNT, causes disrupted spermatogenesis in acrosome formation, resulting in deformed round head spermatozoa [23]. Similarly, hyperactivation of fucosylation‐related genes may result in heavy fucosylation, which impairs male fertility.

#### Hormone dysfunction

Many glycoproteins in the male reproductive system are androgen‐regulated proteins, especially glycan‐modifying enzymes. The activity of fucosyltransferase decreases significantly after antiandrogen therapy, indicating that its expression is associated with sex hormones [59]. In addition, the activities of *α*‐galactosidase and *β*‐galactosidase in spermatozoa are strongly linked to the precesence of gonadotropins in the serum of both fertile male and oligoteratoasthenozoospermia (OAT) patients [53]. In addition to regulating the synthesis and modification of glycoproteins, many reproductive hormones themselves are glycoproteins or regulated by glycoproteins [86]. Human chorionic gonadotropin (hCG) is a glycoprotein highly present in the male genital tract. The alternative glycosylation profile of hCG in seminal plasma from patients with abnormal sperm parameters may suggest pathophysiological roles in spermatogenesis [87]. Sex hormone‐binding globulin carries androgens and estrogen, is expressed in the testis and is involved in acrosome formation during spermatogenesis [88]. Polymorphisms of sex hormone‐binding globulin were shown to affect its serum levels and were associated with male infertility [89].

#### Oxidative stress and environmental impact

It is reasonable that the glycan structures themselves concomitant with the enzymes synthesized, are susceptible to oxidative stresses [90]. For example, clusterin is a known biomarker of oxidative stress [91]. Thus, an imbalanced redox environment may result in altered glycosylation. In addition, a recent study demonstrated that exposure to environmental pollutants may cause alterations in seminal plasma *N*‐glycosylation. In that study, 10 *N*‐glycans, all of which are complex structures, were significantly associated with environmental pollutant exposure, including drinking, smoking, pesticides, air pollution, and photocopying. It is well known that environmental pollutants alter glycosylation. For example, altered *N*‐glycosylation in serum proteins was found to be associated with smoking in lung cancer patients [92]. One of the underlying mechanisms may be that many pollutants are endocrine disruptors that disturb sex hormones, which have a significant impact on glycan‐related enzymes [59]. The above findings extend our understanding of the mechanism by which environmental factors affect male reproduction and reveal specific biomarkers for male infertility.

#### Possible effect of excessive glycation

In addition, glycation, which is a nonenzymatic reaction between sugars and proteins that produces advanced glycation end products, has been found in human spermatozoa and seminal plasma [93]. Glycation differs from glycosylation and is considered detrimental. However, the effect of glycation on male infertility remains unclear [7]. An in vitro study investigating the impact of glycation on sperm function showed that advanced glycation end products could form in whole sperm cells and most significantly in the head region. Although there is no observable damage to sperm motility or hyaluronidase activity, glycation increases the levels of oxidative DNA, which is associated with male infertility in individuals with obesity and diabetes [94]. Additionally, whether excessive glycation disturbs the function of glycoproteins needs further investigation.

#### Infection of male reproductive organs

In addition, infectious diseases of the male reproductive system are considered to cause alterations in glycosylation patterns and are recognized as important factors impairing male fertility potential. Uropathogenic *Escherichia* infection led to hyposialylation in mouse epididymal spermatozoa and epithelial cells. In men with epididymitis, their spermatozoa also showed a lower level of sialylation. The activation of endogenous sialidases may be responsible for this effect. Consequently, this pathological desialylation has adverse effects on spermatozoa against phagocytosis and the complement system [95]. It can be inferred that other epididymis‐related diseases may also trigger similar effects that damage spermatozoa glycosylation patterns, which is worthy of further investigation. In addition, several reports demonstrated changes in the glycome of leucocytospermic patients and reported similar results, showing decreased glycosylation, indicating damage. In leucocytospermic men who suffer from infertility, the fucosylation and sialylation of two glycoproteins, fibronectin, and *α*(1)‐acid glycoprotein, are altered [96]. Moreover, fucosylation of human seminal plasma immunoglobulin G and A secretory components was found to be lower in leucocytospermic patients [97]. The sialylation of the immunoglobulin A secretory component was also found differ between normozoospermic and leucocytospermic seminal plasmas [98]. The potential of glycosylation as a biomarker in seminal plasma indicating male accessory gland inflammation should be highlighted [99].

### Semen glycosylation as a potential biomarker and therapeutic target for male infertility

The presence of specific seminal plasma glycans, glycosylation patterns and glycoproteins have the potential to be a novel biomarker for male infertility, especially idiopathic infertility, which current semen analysis cannot indicate. In particular, the immunomodulatory roles that promote immune privilege in female reproductive organs during fertilization and pregnancy should be highlighted. Many glycoproteins have been used as biomarkers. The potential biomarkers for male infertility (fibronectin [FN], prostatic acid phosphatase [PAP], *β*2‐microglobulin, prolactin inducible protein [PIP], and galectin‐3‐binding protein) are glycosylated [100]. In an earlier study, glycoproteins were isolated on concanavalin A agarose from pooled seminal plasma samples of men with normozoospermia, oligozoospermia and azoospermia. These authors suggested that aminopeptidase N, lactoferrin, PAP, Zn‐a2‐glycoprotein, PSA, glycodelin, Izumo sperm‐egg fusion protein, and PIP are proteins that are differentially expressed in infertile men [101]. However, these glycoproteins were selected as biomarkers based on their protein characteristics, not glycosylation characteristics, and there has been no diagnostic strategy for detecting impaired glycosylation patterns in human semen until now. Recently, changes in clusterin fucosylation have been considered potential biomarkers of male fertility disorders [54, 102]. Reports have demonstrated a significant association between specific seminal plasma *N*‐glycan peaks and sperm quality parameters, including DNA fragmentation and chromatin maturity, although this association could either increase or decrease [103, 104]. It should be noted that abnormal glycosylation‐based diagnosis lacks clinical value because it is used for simply distinguishing asthenozoospermic patients from normozoospermic patients. In some cases, although altered glycosylation was found in patients with abnormal sperm parameters, it is the secondary cause, and multiple abnormalities are common in infertile men. It should be used for the identification of patients in whom abnormal glycosylation is the only or the primary cause of infertility. Therefore, special attention should be given to normozoospermic infertile men. Moreover, these diseases may not be the major cause of fertility but may be a mechanism worth considering. In addition, alterations in glycosylation in the seminal plasma of fertile and infertile men are not significant between the whole glycome and a single glycosylation of selected glycoproteins. The glycomics of seminal plasma or spermatozoa showed substantial intervariability. Considering the relatively high cost of the MS method, a lectin binding assay may still be a feasible strategy to first screen for alterations in the glycosylation pattern of infertile men [82, 97].

### Glycoproteomics in the prostate and other sexual gland diseases

Prostate secretes glycoproteins abundantly. In addition to male infertility, prostate diseases are considered to be closely associated with disturbed protein–carbohydrate interactions and have been the subject of intensive research in recent years [105]. The glycosylation of serum, urine and seminal plasma from prostate disease patients was investigated [106]. First, studies have focused on the different glycoforms of PSA to identify more specific glycoforms for distinguishing benign prostate hyperplasia, indolent prostate cancer and aggressive prostate cancer as biomarkers [107]. For instance, *α*2,3‐sialyl *N*‐glycosylated PSA has potential in the diagnosis of aggressive prostate cancers and is superior to the conventional PSA‐based strategy [108]. After that, the role of glycosylation in the occurrence and development of prostate cancer was revealed. Alterations in both *N*‐glycosylation and *O*‐glycosylation were found to be linked to the development of prostate cancer [109]. In prostate cancer cells, increased sialylation and fucosylation; GlcNAc conjugation; the presence of cryptic and high‐mannose *N*‐glycans; and alterations to proteoglycans were observed. The presence of truncated *O*‐glycans (sTn, sT antigen) was also observed. These alterations could modify many important biological processes in cancer [110].

The other two main accessory sexual glands, the epididymis and seminal vesicles, have received much less attention. An abnormality of the epididymis likely results in a decrease in glycan‐modifying enzymes and impaired sperm maturation. Seminal vesicle‐related diseases may be associated with the secretion of specific glycans and glycoproteins. The decrease in the amount and density of high‐mannose type glycans in seminal plasma may reflect an aberration of the glycosylation or secretion pathway in seminal vesicles [83]. The roles of glycosylation in seminal vesicles should be further explored.

## 4 DEVELOPING SEMEN GLYCOPROTEOMICS

**Figure 3:**
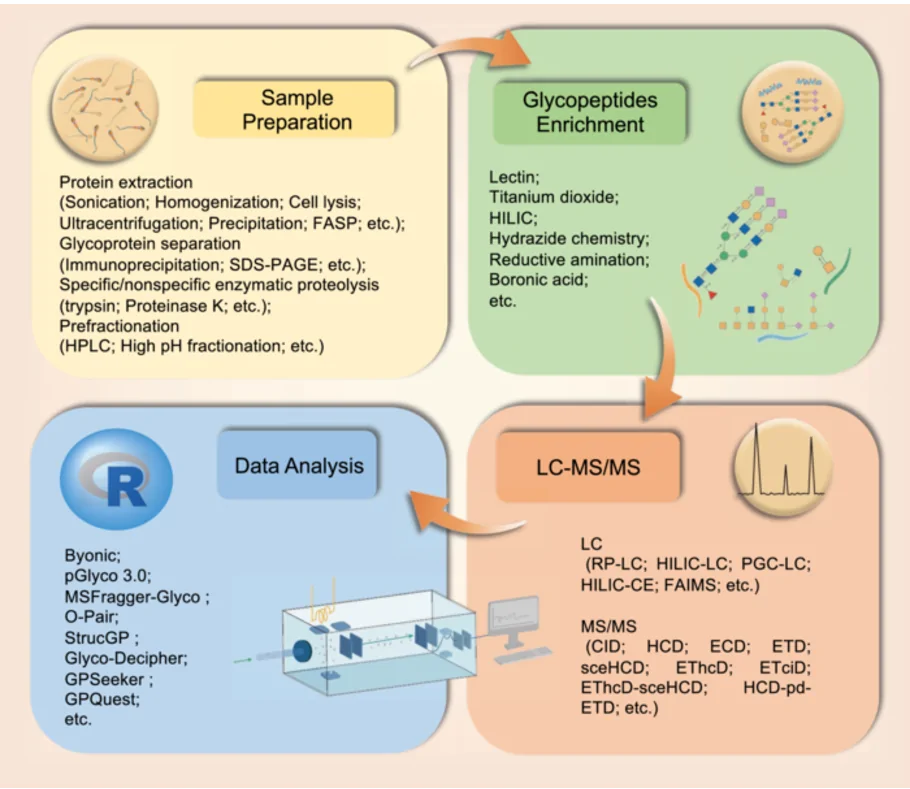
Overview of the methods and approaches of glycoproteomics and applications of human semen. After purification of spermatozoa and seminal plasma from the ejaculate, total proteins are first extracted, and glycoproteins are separated for digestion to obtain glycopeptides. Prefractionation may be needed before glycopeptide enrichment. The most important enrichment strategies included the use of lectin, hydrophilic interaction liquid chromatography (HILIC), and chemical derivatization. LC‐MS/MS, combined with different fragmentation methods, such as high‐energy collision dissociation (HCD), electron capture dissociation (ECD), and electron‐transfer/higher‐energy collision dissociation (EThcD), was then used to characterize the glycopeptide structures. The last step is data analysis, which involves the use of different bioinformatic tools. CID, collision‐induced dissociation; ETciD, electron‐transfer collision‐induced collision dissociation; ETD, electron capture dissociation; FAIMS, field asymmetric ion mobility spectrometry; FASP, filter‐aided sample preparation; HCD‐pd‐ET, higher‐energy collisional dissociation‐product‐dependent electron‐transfer dissociation; HILIC‐CE, hydrophilic interaction liquid chromatography‐capillary electrophoresis; HILIC‐LC, hydrophilic interaction liquid chromatography‐liquid chromatography; HPLC, high performance liquid chromatography; LC, liquid chromatography; LC‐MS/MS, liquid chromatographic‐tandem mass spectrometry; MS/MS, tandem mass spectrometry; PAGE, sodium dodecyl‐sulfate polyacrylamide gel electrophoresis; RP‐LC, reversed‐phase liquid chromatography; sceHCD, stepped collision energy high‐energy collision dissociation; SDS‐PGC‐LC, porous graphitic carbon‐liquid chromatography.

Glycoprotein studies have a history of several decades. In recent years, semen glycosylation studies have been based on the specific binding between glycans and proteins such as antibodies or lectins. However, these traditional strategies are unsatisfactory for understanding the composition and structure of glycans, basic characteristics of protein glycosites, structure‐function relationships and functions of specific glycans on specific proteins [111]. In recent years, with considerable progress in proteomic and glycomic technologies, the compositions, and structures of a variety of glycans, as well as the glycosites on glycoproteins in the male reproductive system, have been identified and functionally characterized [112, 113]. This “true glycoproteomics” approach is used to study intact *N/O*‐glycopeptides (glycopeptides decorated with their native or near‐native *N/O*‐glycoforms) based on advanced mass spectrometry, which can obtain site‐specific glycosylation (glycoprotein, glycopeptide, glycosite, and glycan) information [114]. However, the microheterogeneity, macroheterogeneity, and metaheterogeneity of *N/O*‐glycosylation present great challenges for glycoproteomic analysis [115]. To overcome these difficulties, many methods and approaches have been continuously developed to facilitate the characterization of intact *N/O*‐glycopeptides, and these methods can be divided into four main methods: sample preparation, glycopeptide enrichment, LC‐MS/MS, and data analysis (Figure 3). A few of these methods have been applied in the mapping of the human semen glycoproteome. In addition, improper semen sample processing, which appears in related studies, may have negative effects on glycoproteome results. We believe that the strict quality assurance of semen sample processing combined with the application of novel glycoproteomic techniques will significantly promote human semen glycoproteomic research.

### Glycoproteomic techniques and their applications in human semen

Although great progress has been made in glycoproteomics, these new techniques and methods are rarely applied to the field of male reproduction. Considering that the normal human semen glycoproteome has been established with the aid of a site‐specific intact glycopeptide approach, one of the aims of our analysis is to introduce the newest glycoproteomic strategies, which are expected to have great potential for revealing the roles of glycosylation in the physiological and pathological conditions of male reproduction. Therefore, according to the abovementioned four aspects, we reviewed advanced glycoproteomic workflows and their applications in human semen (Figure 3).

Current sample preparation technologies are capable of handling complex biological samples such as spermatozoa or seminal plasma [116]. The main processes included protein extraction and denaturation, reduction and alkylation, digestion and prefractionation (Figure 3). Standard sample preparation procedures need to be established for different samples to obtain high‐ efficiency protein extraction and a low missed cleavage rate of protein digestion as well as to ensure the repeatability of glycoproteomic studies. For human semen samples, in‐depth sperm protein coverage can be achieved using urea buffer combined with ultrasonication, as reported in our previous study [117]. The glyco‐filter‐aided sample preparation (glyco‐FASP) method was used for the identification of the human spermatozoa glycoproteome [17]. However, when we need to validate a specific glycoprotein or to study a glycoprotein in depth, glycoprotein separation or purification is necessary because of the vast dynamic range of glycoprotein concentrations and the enormous complexity of glycosylation in complex samples. Many methods can be chosen, such as recombinant glycoprotein expression, immunoprecipitation, sodium dodecyl‐sulfate polyacrylamide gel electrophoresis (SDS‐PAGE), and molecular chromatography.

To achieve in‐depth site‐specific glycosylation analysis, selective intact glycopeptide enrichment is one of the most efficient ways to avoid interference from nonglycosylated peptides. Many studies have reviewed these enrichment materials and methods, including lectin, titanium dioxide, hydrophilic interaction liquid chromatography (HILIC), hydrazide chemistry, reductive amination, and boronic acid (Figure 3) [118]. However, each enrichment method or material has its own scope of application. The best choice should be made according to the experimental purpose.

Currently, mass spectrometry combined with various separation methods (reversed‐phase liquid chromatography [RP‐LC], hydrophilic interaction liquid chromatography‐liquid chromatography [HILIC‐LC], porous graphitic carbon‐liquid chromatography [PGC‐LC], hydrophilic interaction liquid chromatography‐capillary electrophoresis [HILIC‐CE], field asymmetric ion mobility spectrometry [FAIMS], etc.) is a powerful tool utilized for intact glycopeptide analysis by different fragmentation methods (collision‐induced dissociation [CID], high‐energy collision dissociation [HCD], electron capture dissociation [ECD], electron capture dissociation [ETD], stepped collision energy high‐energy collision dissociation [sceHCD], electron‐transfer/higher‐energy collision dissociation [EThcD], stepped collision energy high‐energy collision dissociation [ETciD], EThcD‐sceHCD, higher‐energy collisional dissociation‐product‐dependent electron‐transfer dissociation [HCD‐pd‐ETD], etc.) (Figure 3). For instance, collision‐induced dissociation‐tandem mass spectrometry (CID‐MS/MS) was used for profiling the N‐glycoproteome of human seminal plasma, which contains only 50 *N*‐glycoproteins [119]. Parameter setting and fragment mode selection for LC‐MS/MS are key decision points in glycoproteomic experiments [116]. In recent years, we have presented a novel fragmentation method termed EThcD‐sceHCD to improve the performance of intact *N/O*‐glycopeptide analysis. It has been shown to perform better than other methods (sceHCD, EThcD, etc.) in site‐specific glycosylation analysis of many complex clinical samples [120, 121]. Recently, the first *O*‐glycoproteome was mapped by our group via the combination of EThcD‐sceHCD and sceHCD‐MS/MS [21].

Finally, bioinformatic tools are essential for processing intact glycopeptide identification and quantification data. Recent advances in large‐scale glycoproteomics have been reviewed [122]. However, these tools have certain limitations. For example, the lack of multilevel false positive rate (FDR) assessment functions in some software programs will affect the accuracy of identification of intact glycopeptides. Most bioinformatic tools (Byonic, pGlyco, etc.) do not have intact glycopeptide quantitative functions, which limits their clinical application. Some representative software or algorithms and their characteristics are summarized in Table 3.

**Table 3:** Bioinformatic tools for identifying and/or quantifying intact glycopeptide.

Name (references)	*N/O*‐glycans	Glycan ID method	Compatible fragmentation mode	FDR	Free or not	Identification or/and quantification
Byonic [123]	*N/O*	Mass only	Many modes	Peptide	No	Identification
pGlyco2.0 [124]	*N*	Y‐type ions	sceHCD	Peptide + glycan	Yes	Identification
pGlyco3.0 [125]	*N/O*	Y‐type ions	sceHCD; EThcD; ETciD	Peptide + glycan	Yes	Identification and quantification
StrucGP [31]	*N*	Y+B‐type ions	HCD	Peptide + glycan	Yes	Identification
MSFragger‐Glyco [126]	*N/O*	Y+B‐type ions	HCD; sceHCD; HCD‐pd‐ETD	Peptide + glycan	Yes	Identification and quantification
PANDA	‐	‐	‐	‐	Yes	Quantification
pGlycoQuant [127]	‐	‐	‐	‐	Yes	Quantification

Abbreviations: EThcD, electron‐transfer/higher‐energy collision dissociation; ETciD, electron‐transfer collision‐induced collision dissociation; FDR, false discovery rate; HCD, high energy collision dissociation; HCD‐pd‐ETD, higher‐energy collisional dissociation‐product‐dependent electron‐transfer dissociation; sceHCD, stepped collision energy high‐energy collision dissociation.

### Improving quality assurance of semen sample processing

To ensure high‐quality glycoproteome results, proper semen sample processing, which can be affected by many factors, including sample collection, semen examination, and sperm preparation, is needed. However, we noted that the above steps described in semen glycoproteomic studies were ambiguous or even inappropriate, which may have led to low quality assurance. First, information on semen collection, such as masturbation, loss of fraction and sexual abstinence, was not reported in some studies [13, 82]. Second, the semen parameters and fertility status were incomplete; for example, information on sperm morphology and round cells was lacking [17, 18]. Third, there were significant differences in sperm or seminal plasma preparation between the above studies, which may cause contamination and bias in the results (Table 1). In addition to spermatozoa, semen also contains somatic cells (immune cells, epithelial cells), immature spermatogenic cells and cell debris, or other solid components [128]. In this context, filtration should be used to remove jelly‐like granules that do not liquefy and mucus strands. Swim‐up or density gradient centrifugation (DGC) is necessary for purifying spermatozoa unless subsequent glycoproteomic analysis is able to eliminate the effect of nonsperm cells. Moreover, cell debris disturbs the seminal plasma glycoproteome, which needs to be removed using high‐speed centrifugation for a longer time (>10,000*g*) [68]. After careful analysis, we found that most seminal plasma was obtained by low‐speed centrifugation in the above studies, except for [15] and [19]. EVs are still present in seminal plasma even when high‐speed centrifugation is conducted but should not be considered. Therefore, the number, composition and structure of reported glycoproteins might not be accurate. Furthermore, this may contribute to the high overlap between the sperm and seminal plasma glycoproteomes. Improvements in semen sample processing are urgently needed.

**Figure 4:**
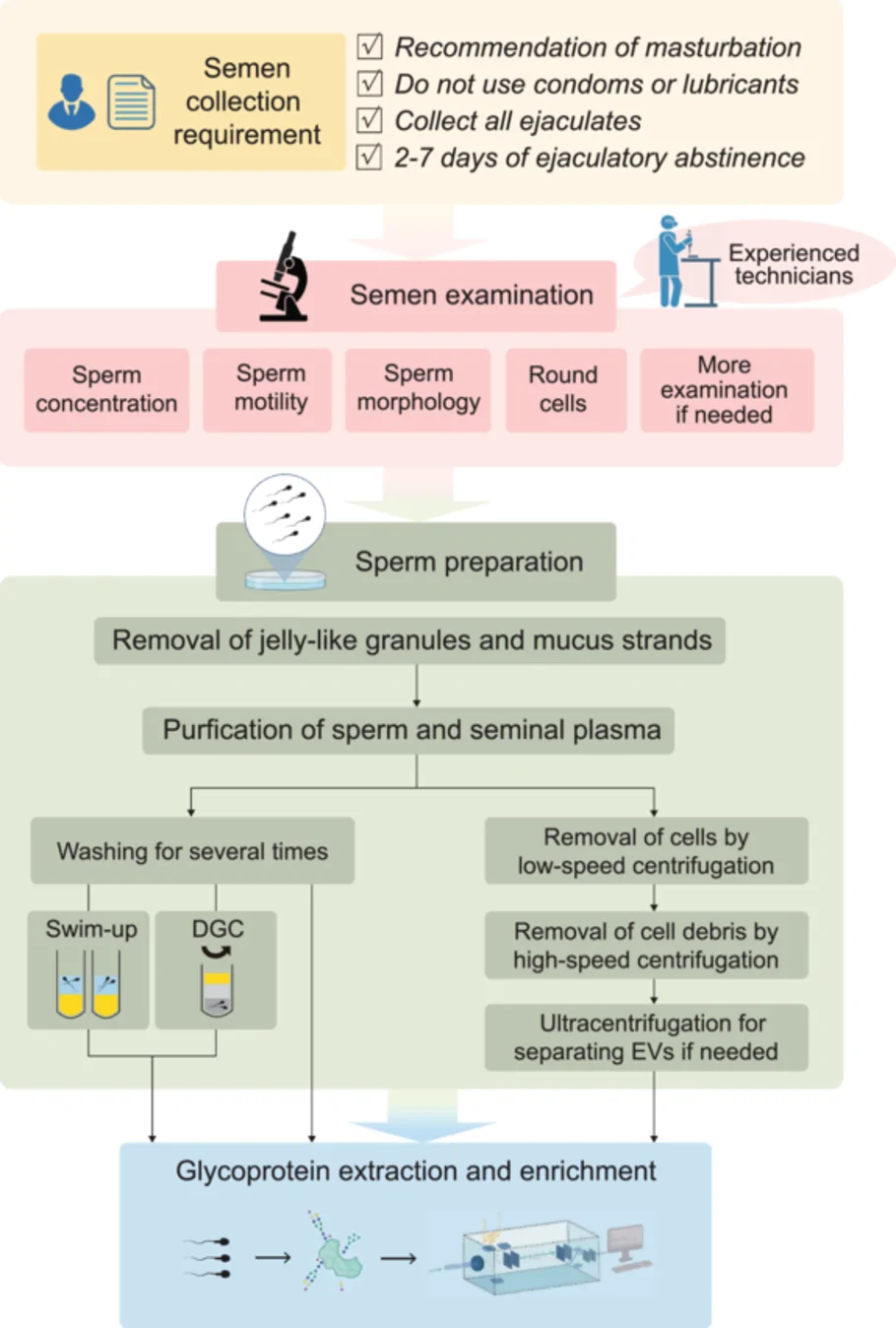
The simplified analytic workflow for semen sample processing to ensure robust and reliable glycoproteomic results. First, the checklist demonstrated the important points that men need to follow during semen collection. After liquification, samples must be analyzed by experienced technicians to evaluate the sperm parameters. For the purification of spermatozoa, swim‐up or density gradient centrifugation (DGC) may be needed to obtain spermatozoa with good quality and total removal of nonsperm cells.

To summarize, we recommend that clear instructions be provided for collecting semen samples from men for glycoproteomic studies. A detailed semen examination should also be conducted by experienced technicians in the andrological laboratory before sample preparation to evaluate whether the sample can be used. After this, the semen samples are subjected to appropriate treatments, such as high‐speed centrifugation, swim‐up or DGC, according to the experimental design, followed by glycoproteomic analysis. Considering that the World Health Organization (WHO) standard may not be easily available in some glycoproteomics laboratories, a simplified workflow for semen sample processing to obtain robust and reliable results is provided in Figure 4.

## 5 FUTURE PERSPECTIVE

### Establishment of the glycoproteome of the whole male reproductive system

The precise *N*‐glycoproteomes of ejaculated spermatozoa and seminal plasma from healthy humans have generally been mapped. In addition to *N*‐glycosylation, *O*‐glycosylation is also highly frequent in the male reproductive tract and is implicated in various essential biological processes, especially immune modulation [9]. For example, truncated *O*‐glycans in seminal plasma glycoproteins are involved in the maternal immune response during fertilization [80]. A higher content of *O*‐linked glycans in spermatozoa has a beneficial effect on fertility and may be attributed to immune escape of neutrophils in the female genital tract [129]. The abovementioned studies revealed several important roles of *O*‐glycosylation, but technique limitations limited the establishment of the *O*‐glycosylation profile of human semen. This limitation is expected to be overcome based on a novel strategy termed GlycoTCFM developed by our group and by using this strategy, we profiled the most comprehensive *O*‐glycoproteome map of human semen [21, 130]. Furthermore, the *O*‐glycoproteome profile of human semen has been continuously updated and will largely extend the human semen glycoproteome for clinical application in the future.

Glycosylation continues throughout the lifetime of sperm. Key events for sperm fertility competence probably occur in any period, and the sperm expelled from the body may not be captured. Apparently, the glycoproteome of the ejaculated spermatozoa and seminal plasma represents the tip of the iceberg. Many studies have illustrated changes in glycosylation patterns among testicular, epididymal, ejaculated, and capacitated sperm. The quantitative assessment of spermatogenesis by histochemistry is important for identifying the types of spermatogenic cells and determining the presence or absence of disorders [131]. Nevertheless, a precise glycoproteome of spermatogenesis, maturation, capacitation, and fertilization is needed, which could help us to understand the complex, dynamic, spatiotemporal regulation of glycosylation in male reproduction in detail. Additionally, the glycosylation profiles of the secret from accessory sexual glands should be improved, which will deepen our knowledge of the role of these secretions in male reproductive health. Similarly, a recent review summarizing protein glycosylation in human maternal‐fetal crosstalk highlighted its involvement in the regulation of early pregnancy, which also demonstrated the importance of constructing a complete glycosylation profile for male reproduction [132].

### Potential of glycoproteomics for clinical use in male reproduction

#### Development of novel biomarkers and therapeutic targets for male reproductive health

A variety of sperm parameters, such as concentration, motility, morphology, genetic damage, mitochondrial function, and the acrosome reaction, are used in clinical practice. Undeniably, these laboratory tests are capable of diagnosing and guiding the treatment of patients suffering from infertility. However, a substantial proportion of these patients present normospermia and are often categorized as idiopathic male infertility. Recent studies revealed that normospermic infertile patients, although they produce normal testicular spermatozoa, may exhibit abnormalities in sperm maturation, capacitation, immunoprotection, and recognition of oocytes, but effective clinical laboratory tests are lacking. Aberrant changes in glycosylation have been linked to the abovementioned male infertility and have the potential to be novel biomarkers. Nonetheless, few glyco‐biomarkers, regardless of the presence of glycans or glycoproteins, have been developed for clinical use.

Glycan‐modifying enzymes have great potential as biomarkers of epididymal function for diagnosing impaired sperm maturation. It should be noted that a study screened 65 enzymes from the seminal plasma of individuals with azoospermia and found that α‐glucosidase is the only enzyme showing variation [133]. Further study proved that the neutral form of α‐glucosidase in the seminal plasma was specifically expressed in the epididymis rather than other acid isoenzymes from the prostate [134]. Thus, it has been the only glycosylation‐related enzyme for clinical use in seminal biochemical assays for more than 30 years [135]. However, despite being considered a marker of epididymal function, the clinical use of neutral α‐glucosidase in the diagnosis of obstructive azoospermia is limited [136]. A decrease in neutral α‐glucosidase suggested compromised sperm maturation. However, it may not be the key enzyme participating in this process [55, 137]. In addition, altered levels of many acid glycosidases are involved in male infertility. Not only their decrease but also their increase may play a role in the soluble and nonsoluble fractions of sperm and seminal plasma in oligoasthenoteratozoospermia [53]. In addition to the use of glycan‐related enzymes from seminal plasma for detecting impaired sperm maturation, glycan and glycoprotein abnormalities in spermatozoa and seminal plasma are considered biomarkers of sperm capacitation, sperm‐egg binding, immune‐related infertility, and sexual glands. Notably, there are no effective biomarkers for evaluating these parameters. For example, for biomarkers specific to seminal vesicles, there is only one marker, fructose, detected in the clinic, although it is mainly used for obstructive azoospermia. However, a crucial glycoprotein in fertility, glycodelin S, is secreted by seminal vesicles and released in the seminal plasma. Glycosylation impacts sperm capacitation and may influence seminal vesicle diseases [81]. Capacitation‐associated glycans and glycoproteins in the sperm head, such as mannosylation, may become candidates for evaluating sperm capacitation [138, 139]. Furthermore, changes in spermatozoa and seminal plasma glycosylation patterns are promising for the diagnosis of immune‐related infertility. It should be noted that many single glycans, glycoproteins, and rough glycosylation patterns have been identified as potential biomarkers, but they are likely relatively ineffective due to the high complexity of glycosylation in male reproduction. Therefore, the recently established precision glycoproteome of human semen and seminal plasma enables us to screen the most suitable biomarkers or construct a panel as well as a model for more accurate and specific diagnosis of male infertility.

#### Help improve assisted reproductive technology (ART)

Mapping the precise glycoproteome of human spermatozoa will have a favorable effect on ART even under intracytoplasmic sperm injection (ICSI) conditions. Sperm cryopreservation is a commonly used strategy for patients receiving ART and inevitably has a detrimental influence on sperm. The prediction of freeze recovery has clinical significance for patients, and knowledge of glycosylation on the sperm surface may shed light on this topic. Lectin microarray tests of ejaculates with different freezability revealed that *Agaricus bisporusagglutinin* (ABA) has the potential to be a biomarker of freeze tolerance [140]. In addition, the degree of sperm glycosylation may impact the process of sperm‐selection during ART [141]. The protection of glycoproteins during sperm cryopreservation results in the improvement of thawed sperm [142]. The effect of seminal plasma on the glycosylation profile of cryopreserved sperm should be further investigated [143]. Moreover, changes in glycosylation on the sperm surface may occur in the treatment of sperm for ICSI. For example, *L*‐carnitine and pentoxifylline treatment were shown to impact glycoconjugates on the surface of testicular sperm. In summary, the sperm glycoproteome provides information that helps determine ART strategies and improve sperm freezing, sperm selection and sperm preparation procedures.

## 6 CONCLUSION

While the investigation of glycosylation in male reproduction has a long history, recent studies have elaborated on the precise site‐specific glycosylation in human ejaculated spermatozoa and seminal plasma. Semen contains several distinctive glycosylations, particularly extremely high fucosylation, which requires further in‐depth studies. The glycoproteome plays a vital role in immunomodulation, capacitation, and sperm‐oocyte binding. Furthermore, alterations in the glycosylation profile of testicular, epididymal, and capacitated sperm may be closely linked to glycan‐modifying enzymes and EVs. Male infertility is often associated with changes in semen or seminal plasma, which can lead to dysregulation of the aforementioned processes. Glycosylation abnormalities may result from genetic, endocrine, oxidative stress, or infection, and could explain some cases of idiopathic male infertility. However, the use of glycoproteins as biomarkers in male reproductive diseases is still understudied. This paper also discusses advanced glycoproteomic techniques that scientists can use to address current challenges in male reproduction. Breakthroughs in *O*‐glycosylation research have revealed the complex, dynamic, and spatiotemporal regulation of the entire process of sperm development. This summary confirms the significance of glycans, glycan patterns, and glycoproteins as biomarkers for the diagnosis of male reproductive disorders and highlights their value in assisted reproductive technology, such as sperm cryopreservation and selection.

## AUTHOR CONTRIBUTIONS

**Qingyuan Cheng**: Writing—original draft. **Mengqi Luo**: Writing—original draft. **Zihe Xu**: Writing—original draft. **Fuping Li**: Conceptualization. **Yong Zhang**: Conceptualization; writing—review and editing.

## CONFLICT OF INTEREST STATEMENT

The authors declare no conflict of interest.

## ETHICS STATEMENT

No animals or humans were involved in this study.

## Data Availability

No new data and scripts were used for this analysis. Supporting Information (graphical abstract, slides, videos, Chinese translated version, and update materials) is available online at DOI or http://www.imeta.science/imetaomics/.
